# Impact of malnutrition on survival in adult patients after elective cardiac surgery: Long-term follow up data

**DOI:** 10.1016/j.dib.2020.106651

**Published:** 2020-12-17

**Authors:** Sergey M. Efremov, Tatiana I. Ionova, Tatiana P. Nikitina, Pavel E. Vedernikov, Timur A. Dzhumatov, Timofey S. Ovchinnikov, Abduvahhob A. Rashidov, Alexandr E Khomenko, Christian Stoppe, Daren K. Heyland, Vladimir V. Lomivorotov

**Affiliations:** a Department of Anesthesiology and Intensive care, Saint Petersburg State University Hospital, St. Petersburg, Russian Federation; b Quality of Life and Monitoring Unit, Saint Petersburg State University Hospital, St. Petersburg, Russian Federation; c Department of Anesthesiology and Intensive care, E. Meshalkin National Medical Research Center, Novosibirsk, Russian Federation; d Medical Faculty, Saint Petersburg State University, St. Petersburg, Russian Federation; e Department of Intensive Care Medicine, Uniklinik RWTH Aachen University, Germany; f Department of Critical Care Medicine, Queen's University, Kingston, Ontario, Canada; g Novosibirsk State University, Novosibirsk, Russian Federation

**Keywords:** Malnutrition, Cardiac surgery, Survival, Nutritional screening, Albumin

## Abstract

The data article refers to the paper titles “Impact of malnutrition on long-term survival in adult patients after elective cardiac surgery” [1]. The data refer to the analysis of the relationship between baseline malnutrition and long-term mortality after cardiac surgery. Baseline demographic, nutritional, and medical history data were collected for each enrolled patient. Baseline serum albumin and C-reactive (CRP) protein levels were also obtained. Surgical risk was assessed in accordance with the logistic EuroSCORE. Intraoperative data including cardiopulmonary bypass (CPB) time and postoperative characteristics, such as postoperative complications, number of days in the ICU, and hospitalization duration, were also collected. Data on nutritional status were collected using four nutritional screening tools: (1) malnutrition universal screening tool (MUST), (2) short nutritional assessment questionnaire (SNAQ), (3) mini-nutritional assessment (MNA), and (4) nutritional risk screening 2002 (NRS-2002). Both electronic medical records and phone interviews were used for survival data collection. ROC analysis was performed to analyze prognostic value of baseline and perioperative variables on long-term mortality. Univariate and multivariate logistic regression analysis of predictors of 3- and 8-year mortality were performed. Kaplan-Meyer curves, describing the impact of baseline and perioperative characteristics on 3- and 8-year survival were also performed.

**Specifications Table**

**Table:** 

Subject	Cardiology and Cardiovascular Medicine
Specific subject area	Nutrition in cardiology
Type of data	Tables: 2 Figures: 17
How data were acquired	Nutritional screening was performed by trained study personal in the preoperative period within 48 h of admission to the hospital. Characteristics of perioperative course were extracted from medical records. Both electronic medical records and phone interviews were used for survival data collection.
Data format	Raw
Parameters for data collection	Data from patients meeting inclusion/exclusion criteria for prospective observational study (NCT01366807) were collected.
Description of data collection	Data were collected by physical exam, phone interviews and extracted from medical records by trained study personal.
Data source location	Institution: E. Meshalkin National Medical Research Center City/Town/Region: Novosibirsk Country: Russian Federation Repository name: Mendeley Data
Data accessibility	Row data are hosted in the trusted repository. Repository name: Mendeley Data Data identification number: 10.17632/2m6yxvr23h.3 Direct URL to data: https://data.mendeley.com/datasets/2m6yxvr23h/3
Related research article	Efremov S, Ionova T, Nikitina T, Vedernikov P, Dzhumatov T, Ovchinnikov T, Rashidov A, Stoppe C, Heyland D, Lomivorotov V. Impact of Malnutrition on Survival in Adult Patients After Elective Cardiac Surgery, Nutrition, In Press https://doi.org/10.1016/j.nut.2020.111057

## Value of the Data

• Data describe the impact of malnutrition and other baseline and perioperative parameters on long-term survival among cardiac patients.
• Researchers conducting studies on nutrition in cardiology may benefit from these data.
• Data may be used for sample size calculation and estimation of clinically relevant end points in future clinical trials.

## 1 Data Description

Raw data:

(A) Patient ID;
(B) Age in years;
(C) Gender, [1] male, [2] female;
(D) Primary pathology: [1] coronary artery disease, [2] heart valve disease;
(E) Primary diagnosis: [1] coronary artery disease, [2] mitral stenosis, [3] mitral regurgitation, [4] aortic stenosis, [5] aortic regurgitation, [6] tricuspid regurgitation, [7] pathology of aorta, [8] pathology of pulmonary artery;
(F) Type of surgery: [1] coronary artery bypass grafting, [2] valve surgery (repair or prothesis), [3] combined surgery (CABG and valve surgery), [4] aortic surgery;
(G) Redo surgery: [1] history of open hert surgery;
(H) Diabetes: [1] history of type 1 or 2 diabetes mellitus at the enrollment;
(I) NYHA class: class of heart failure with accordance to New York Heart Association graded 1 to 4;
(J) Logistic Euroscore: predicted mortality with accordance to Logistic Euroscore, percent;
(K) SNAQ: results of nutritional screening with accordance to Short Nutritional Assessment Questionnaire where 0 is no malnutrition, 1 is moderate malnutrition and 2 is severe malnutrition;
(L) MUST: results of nutritional screening with accordance to Malnutrition Universal Screening Tool where 0 is no risk of malnutrition, 1 is medium risk of malnutrition and 2 is high risk of malnutrition;
(M) MNA: results of nutritional screening with accordance to Mini Nutritional Assessment screening tool where 0 is no risk of malnutrition, 1 is at risk of malnutrition and 2 indicates malnutrition;
(N) NRS_2002: results of nutritional screening with accordance to Nutritional Risk Screening 2002 tool where 0 is no malnutrition and 1 is malnutrition;
(O) SGA_grade: results of nutritional assessment with accordance to Subgective Global Assessment where 0 is no malnutrition, 1 is suspected or moderate malnutrition and 2 is severe malnutrition;
(P) BMI: body mass index expressed in kg/m^2^;
(Q) Preoperative total lymphocyte count in peripheral blood, expressed in cells/microliter;
(R) Preoperative total blood protein, expressed in g/L;
(S) Preoperative blood albumin, expressed in g/L;
(T) Preoperative thrombocytes (platelets), expressed in number⋅10^9^/L;
(U) C reactive protein in blood, expressed in mg/L;
(V) Baseline left ventricle ejection fraction, expressed in percent;
(W) Cardiopulmonary bypass time during surgery, expressed in minutes;
(X) Aortic cross clamp time during surgery, expressed in minutes;
(Y) Ventilation is mechanical ventilation time after surgery, expresses in hours;
(Z) Any postoperative complications: [1] development of any complication (columns AA-AH) after surgery during hospitalization;
([) New dialysis: [1] development of acute kidney failure required new dialysis after surgery during hospitalization;
(\) (AB) Delirium: [1] development of postoperative delirium during hospitalization;
(]) (AC) Inotropes: [1] postoperative requirement of inotropes (catecholamines) during hospitalization;
(^) (AD) Cardiac arrhythmia: [1] postoperative development of new onset atrial fibrillation or bradycardia required temporary peacemaking during hospitalization;
(_) (AE) Bradycardia: [1] development of postoperative bradycardia required temporary peacemaking during hospitalization;
(`) (AF) New onset atrial fibrillation: [1] postoperative development of new onset atrial fibrillation during hospitalization;
(a) (AG) Bleeding : [1] clinically relevant bleeding, required intervention (re exploration, fresh frozen plasma administration etc.) after surgery during hospitalization;
(b) (AH) Readmission to ICU: [1] readmission to intensive care unit during the initial hospitalization by any reason;
(c) (AI) Intensive care unit stay expressed in days;
(d) (AG) Hospitalization is duration of hospital stay during initial hospitalization, days;
(e) (AK) In hospital mortality: [1] death during initial hospitalization;
(f) (AL) Survival: total number of days alive after surgery, [lost] means patient was lost to follow up;
(g) (AM) Death: [1] patient died during the follow up period, [lost] patient was lost to follow up.

Repository name: Mendeley Data

Data identification number: 10.17632/2m6yxvr23h.3

Direct URL to data: https://data.mendeley.com/datasets/2m6yxvr23h/3

The tables and figures are supplementary data associated with the research paper entitled “Impact of Malnutrition on Survival in Adult Patients After Elective Cardiac Surgery” Nutrition, In Press https://doi.org/10.1016/j.nut.2020.111057. The aforementioned paper investigated the relationship between malnutrition and long-term survival in patients operated on under cardiopulmonary bypass (CPB).

ROC analyses were performed to determine the predictive values and the cut-off points of studied variables. Values obtained from ROC analysis of 8 years follow-up stratified by mixed, coronary artery disease (CAD) and heart valve disease (HVD) cohorts are reported in Table 1. Youden's J statistic was determined for every represented variable. Characteristics for cut-off points are specificity and sensitivity. Corresponding values of variables are represented in the “Criterion” column. AUC values and p-value for obtained ROC curves were also computed.

**Table 1:** presents values obtained from ROC analysis of 8 years follow-up stratified by mixed, HVD and CAD cohort. For every represented variables Youden J statistic was determined. Characteristics for cut-off points are specificity and sensitivity. Corresponding values of variables are represented in column “Criterion”. Values of AUC and p-value for obtained ROC curves were also computed.

Variable	Specificity	Sensitivity	Criterion	AUC	p
**Mixed cohort**					
Albumin	0.73	0.52	42.5	0.64	<0.001
CRP	0.49	0.67	1.85	0.586	0.032
CPB time	0.73	0.49	111.5	0.632	<0.001
Aortic cross clamp	0.67	0.5	73.5	0.598	<0.001
Thrombocytes	0.61	0.5	210.5	0.539	0.17
LVEF	0.68	0.46	57.75	0.549	0.101
Age	0.53	0.64	59.5	0.605	<0.001
**HVD patients**					
Albumin	0.68	0.55	42.5	0.627	0.002
CRP	0.58	0.7	2.25	0.661	<0.001
CPB time	0.55	0.66	111.5	0.606	0.005
Aortic cross clamp	0.65	0.51	96.5	0.569	0.067
Thrombocytes	0.63	0.52	199.5	0.555	0.147
LVEF	0.73	0.38	57.75	0.535	0.368
Age	0.69	0.46	62.5	0.579	0.035
**CAD patients**					
Albumin	0.78	0.48	42.5	0.65	0.002
CRP	0.37	0.9	3.85	0.592	0.191
CPB time	0.36	0.88	56.5	0.646	<0.001
Aortic cross clamp	0.49	0.73	39.5	0.614	0.009
Thrombocytes	0.66	0.43	252.5	0.5	0.992
LVEF	0.63	0.61	57.5	0.616	0.017
Age	0.51	0.76	59.5	0.664	<0.001

CRP – C-reactive protein, CPB – cardiopulmonary bypass, LVEF – left ventricle ejection fraction.

**Table 2:** presents values of predictors obtained by Cox proportional hazard model for 8 and 3 years follow-up. Column “Characteristic” denotes a name of predictor, “HR” - is a hazard ratio, “95% CI” - is a 95% confidence interval for value of HR, “p-value” - measure of significance for HR.

	Univariate models			Full mixed Cox model			Optimal mixed Cox model		
Characteristic	HR	95% CI	p	HR	95% CI	p	HR	95% CI	p
**8 years follow-up Cox regression models**									
Logistic Euroscore	1.05	1.03, 1.07	<0.001	1.03	1.00, 1.08	0.080	1.05	1.03, 1.08	<0.001
MUST > 0	1.38	0.89, 2.15	0.2	1.14	0.61, 2.13	0.7			
CPB > 111.5	2.44	1.70, 3.50	<0.001	1.78	1.01, 3.14	0.045	1.95	1.27, 2.99	0.002
CRP > 1.85	1.85	1.10, 3.12	0.021	1.58	0.90, 2.77	0.11			
Albumin > 42.5	0.34	0.22, 0.51	<0.001	0.55	0.31, 0.98	0.041	0.42	0.27, 0.65	<0.001
**3 years follow-up Cox regression models**									
Logistic Euroscore	1.06	1.04, 1.09	<0.001	1.03	0.97, 1.08	0.306	1.06	1.02, 1.10	<0.001
MUST > 0	1.80	1.03, 3.12	0.038	1.39	0.62, 3.11	0.429			
CPB > 119	2.58	1.58, 4.20	<0.001	2.13	1.00, 4.53	0.051	2.15	1.19, 3.88	0.012
CRP > 3.05	2.94	1.47, 5.87	0.002	2.65	1.22, 5.75	0.013			
Albumin > 42.5	0.25	0.14, 0.45	<0.001	0.34	0.16, 0.75	0.007	0.32	0.18, 0.59	<0.001

MUST – malnutrition universal screening tool, CPB – cardiopulmonary bypass, CRP – C-reactive protein.

**Fig. 1:**
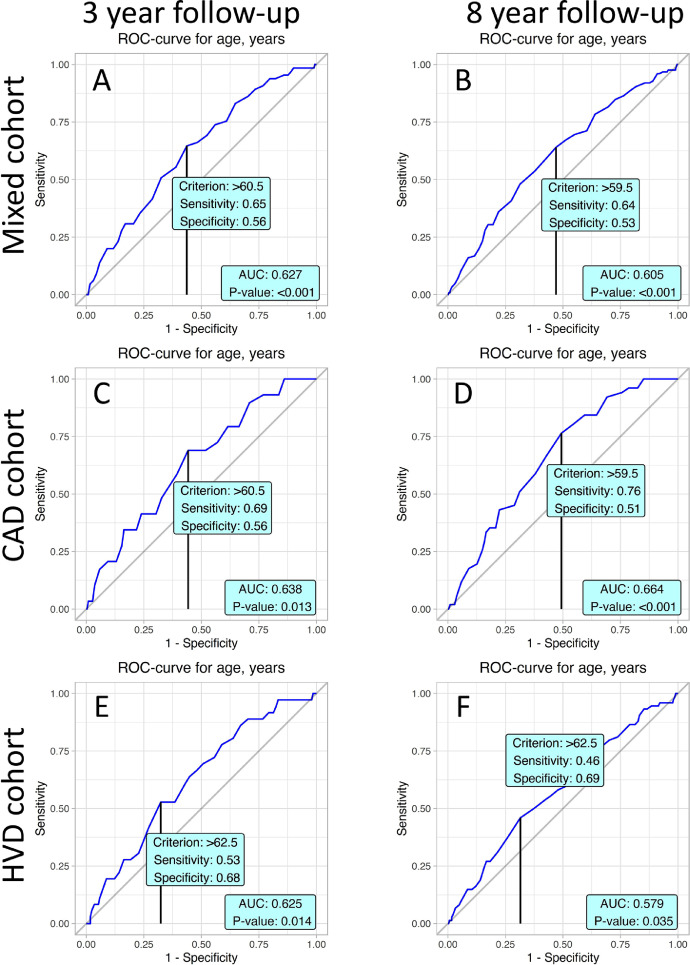
According to ROC-analysis age is a significant risk factor of occurrence of the event considering by cohort and time of follow-up.

The following risk factors of mortality in accordance with the cohort and follow-up periods were analyzed: age, serum albumin, CPB time, aortic cross-clamp time, C-reactive protein, and left ventricle ejection fraction. Thus, ROC analysis revealed the corresponding cut-off values of age for 3- and 8-year follow-up periods in studied patient cohorts (Fig. 1).

**Fig. 2:**
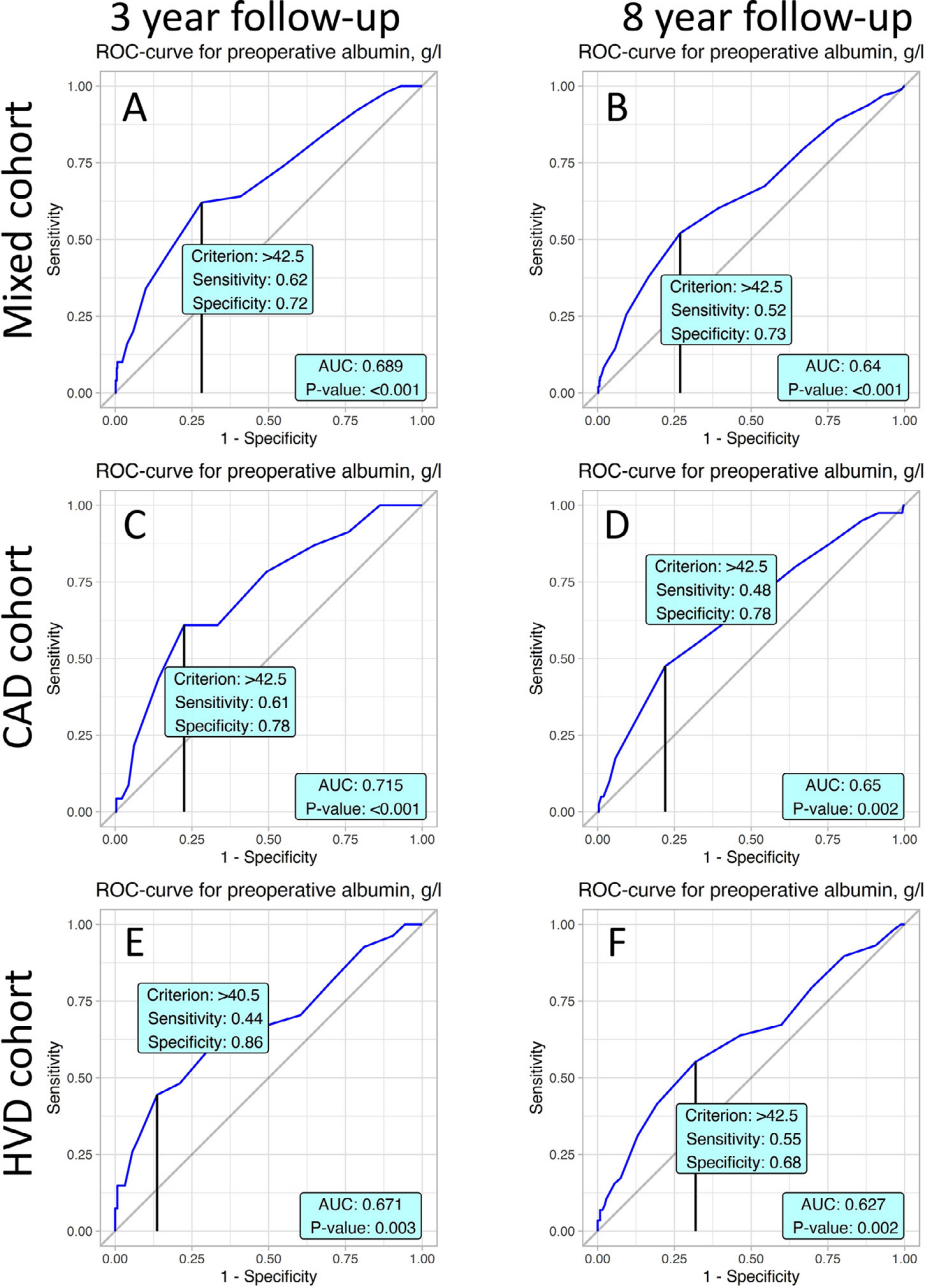
ROC analyses present prognostic value of albumin in postoperative mortality with accordance to studied cohorts and follow-up periods.

For every follow-up and all cohorts, preoperative albumin level has a value on a ROC-curve that has significant AUC. Thus, for all cohorts and follow-up periods, this cut-off value is 42.5 g/l except for 3-year follow-up in the HVD cohort, which is 40.5 g/l (Fig. 2).

**Fig. 3:**
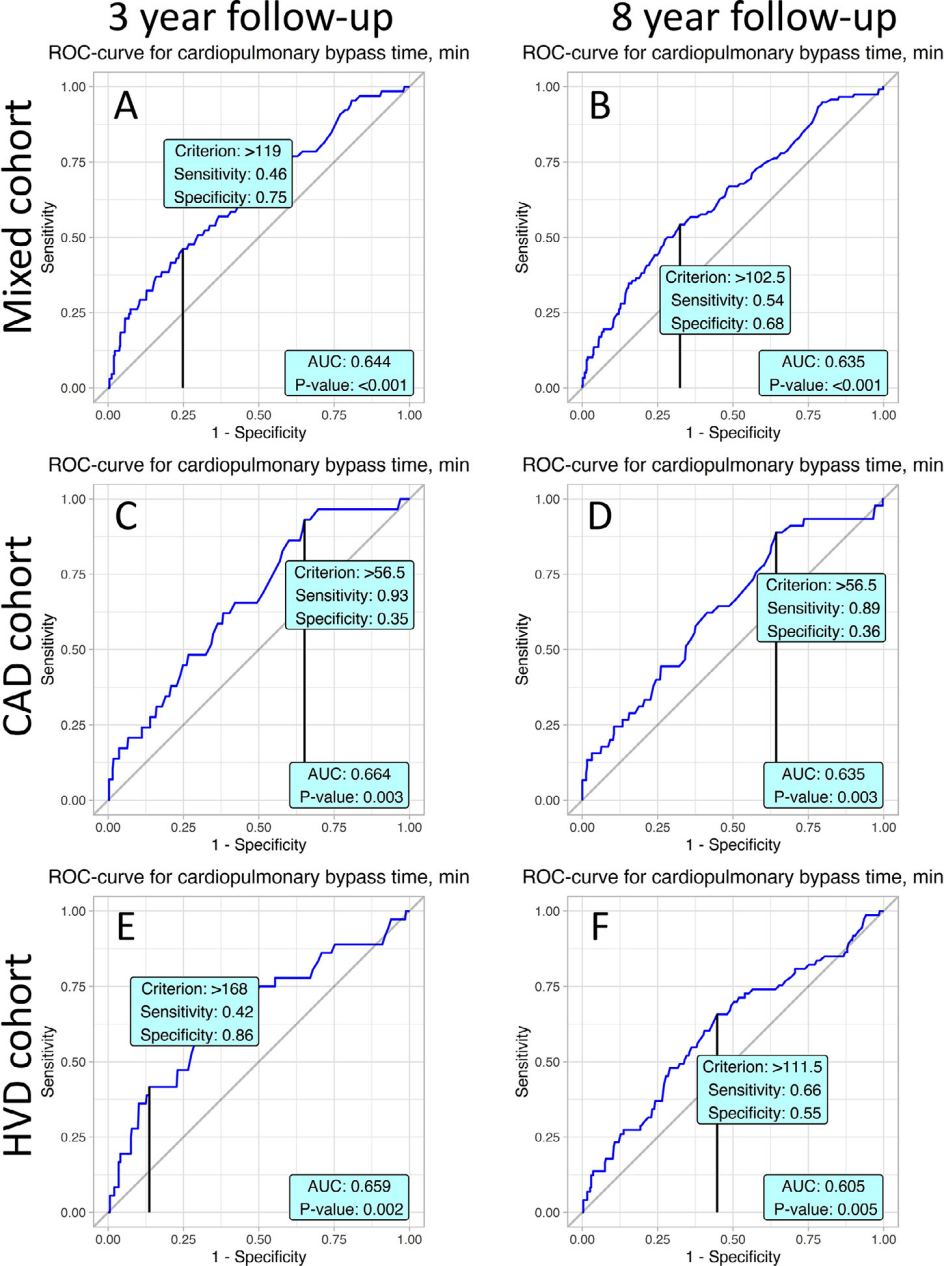
ROC curves present prognostic value of cardiopulmonary bypass time in postoperative mortality with accordance to studied cohorts and follow-up periods.

Predictive values of CPB time in all studied periods and cohorts are presented in Fig. 3. They differ vastly, from 56.5 min in the CAD cohort in the 3-year follow-up (Fig. 3C) to 168 min in the HVD cohort in the 3-year follow-up (Fig. 3E).

**Fig. 4:**
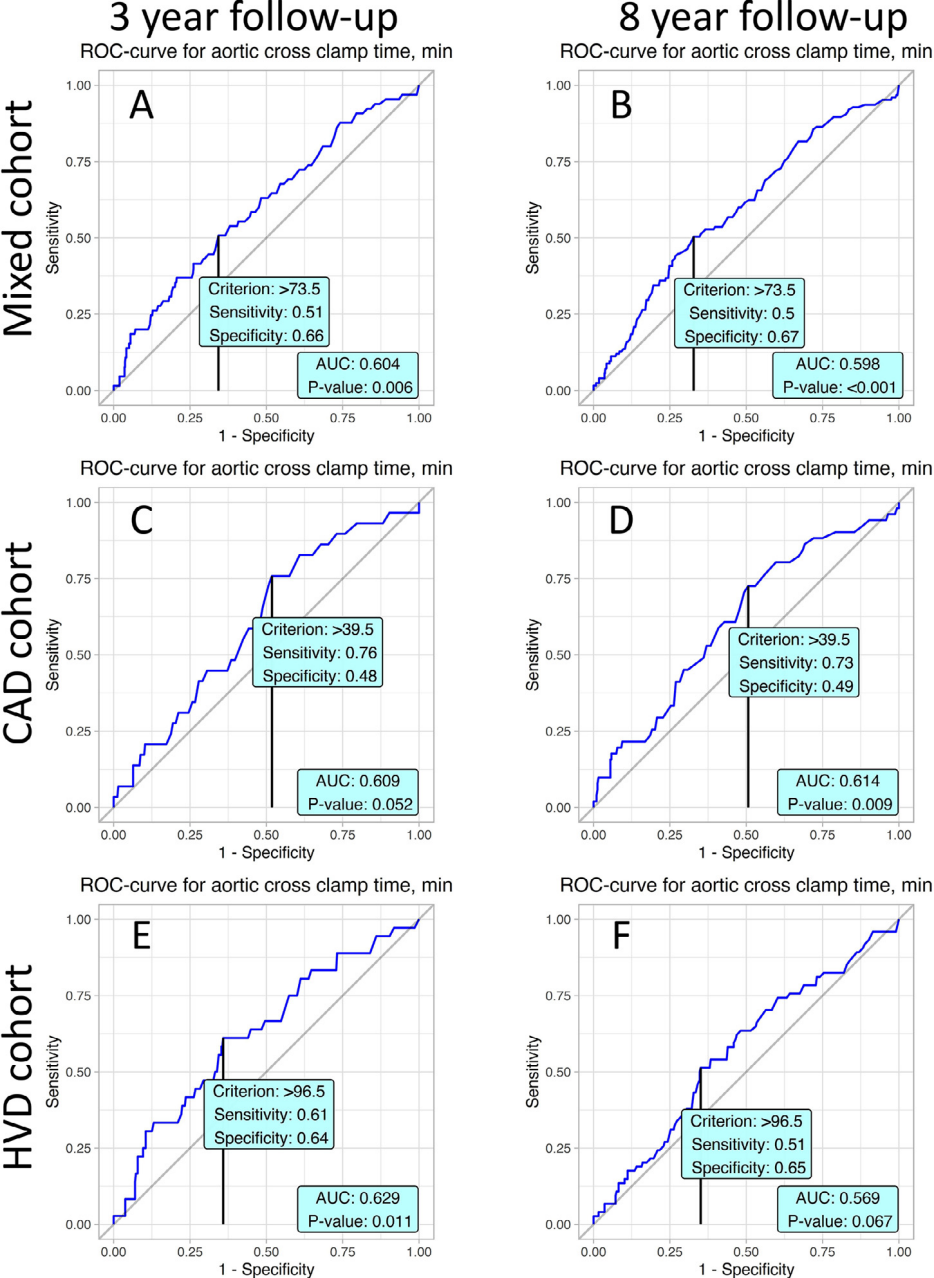
ROC curves present prognostic value of aortic cross clamp time in postoperative mortality with accordance to studied cohorts and follow-up periods.

ROC curves of predictive value of aortic cross-clamp time are presented in Fig. 4. Significant cut-off values are averaged from 39.5 min (Fig. 4D) to 96.5 min (Fig. 4F).

**Fig. 5:**
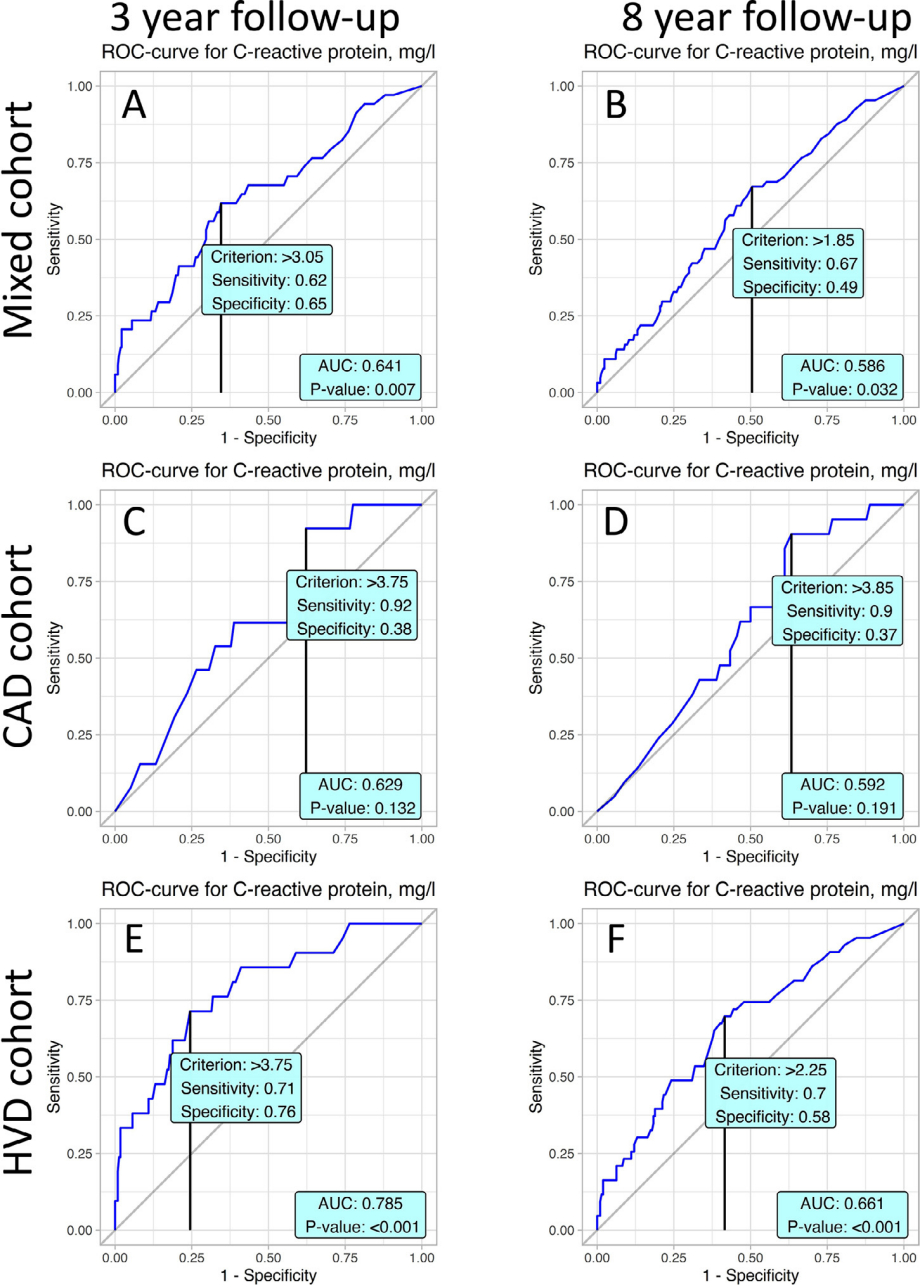
ROC curves present prognostic value C-reactive protein level in postoperative mortality in accordance to studied cohorts and follow-up periods.

Significant cut-off values of C-reactive protein levels were found in all follow-up periods among mixed and HVD cohorts (Fig. 5).

**Fig. 6:**
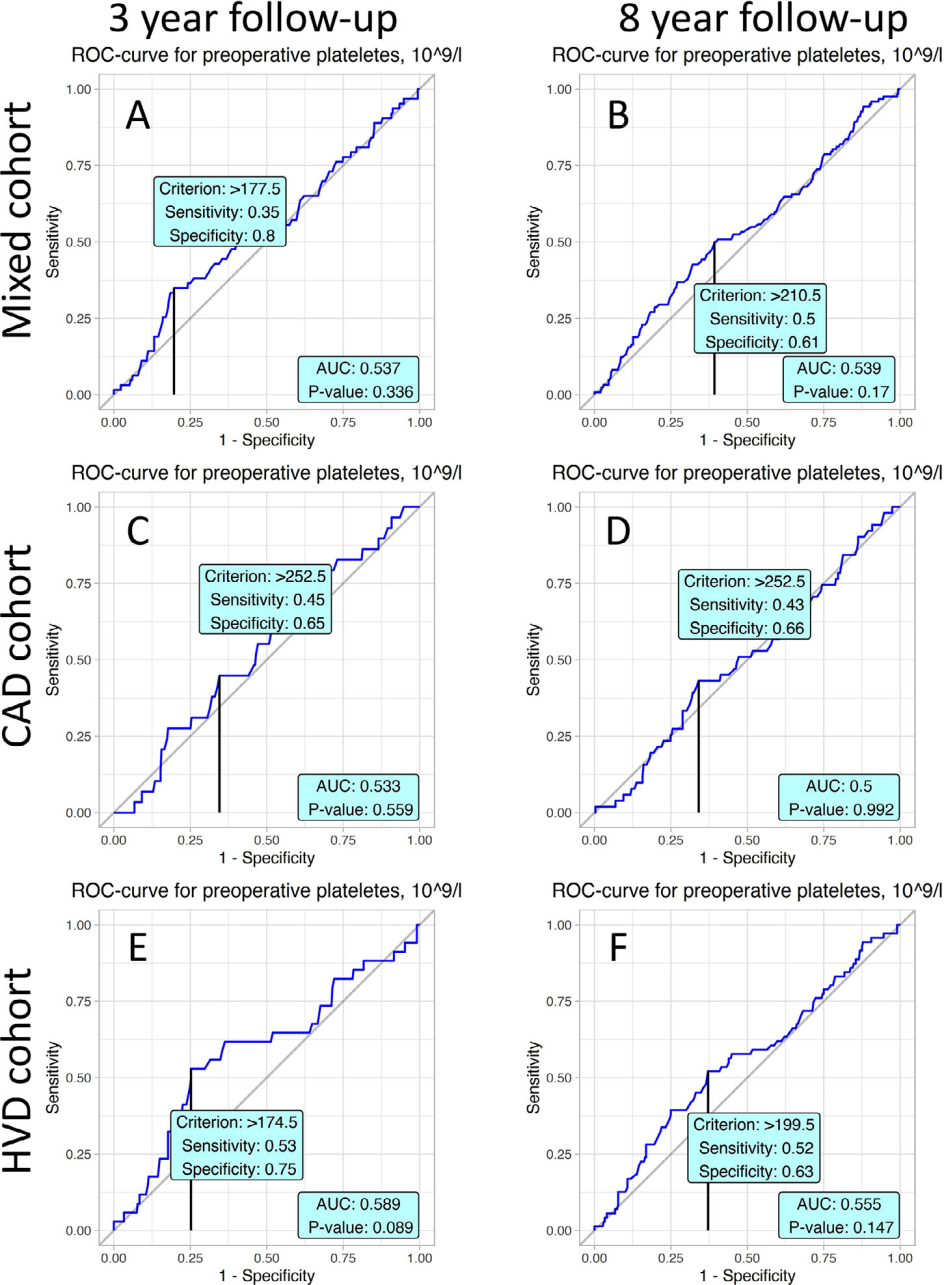
ROC curves present prognostic value baseline platelets of peripheral blood in postoperative mortality in accordance to studied cohorts and follow-up periods.

Absence of predictive value of preoperative platelets level is illustrated in Fig. 6.

**Fig. 7:**
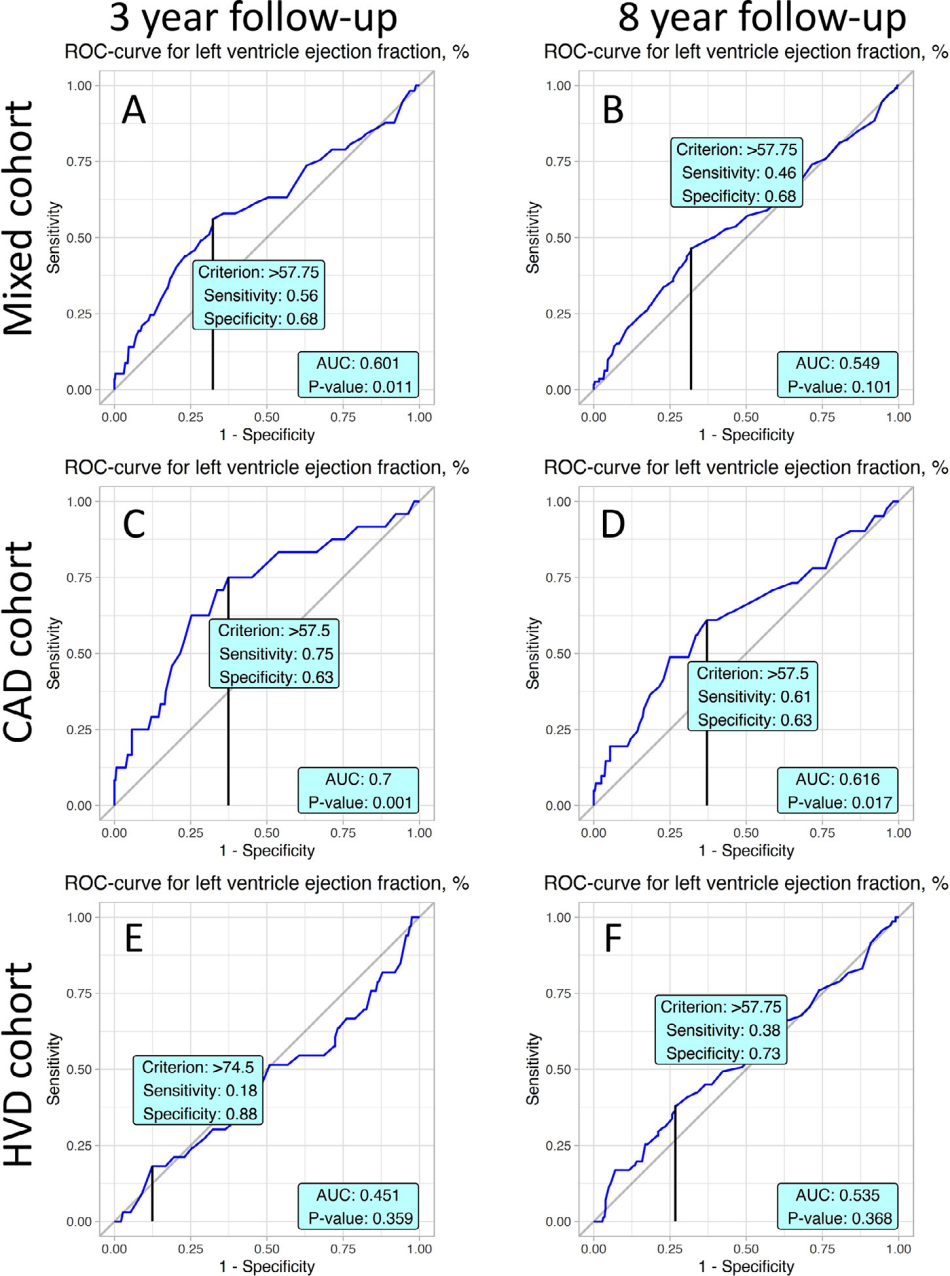
ROC curves present prognostic value of baseline left ventricle ejection fraction in postoperative mortality in accordance to studied cohorts and follow-up periods.

Left ventricle ejection fraction has significant cut-off values for both time periods in the mixed cohort and 3-year time period in the CAD cohort (Fig. 7).

**Fig. 8:**
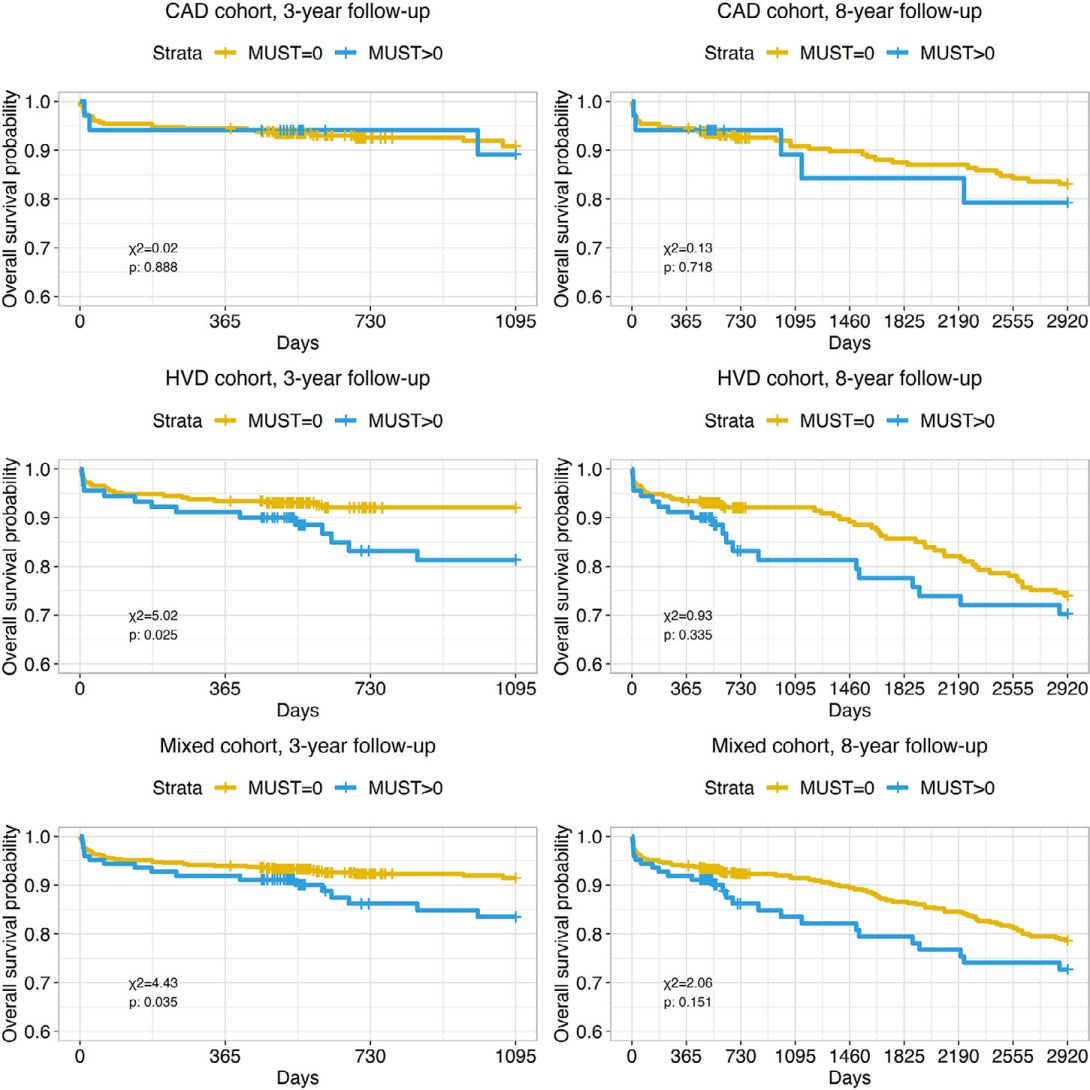
Kaplan-Meyer curves present impact of malnutrition with accordance to Malnutrition Universal Screening Tool (MUST) on long-term mortality in studied cohorts and follow-up periods.

Kaplan-Meyer survival analysis was performed to investigate the impact of preoperative and perioperative characteristics on long-term survival. Analysis of impact of malnutrition on 3- and 8-years survival was performed separately for the cohort of patients with CAD, HVD, and mixed cohort. Malnutrition was a significant predictor for survival in the HVD cohort (*n* = 379) and mixed cohort (*n* = 738) only in the 3-year follow-up (*p* = 0.025 and *p* = 0.035, respectively). During the 8-year follow-up, differences in survival probability were not found (Fig. 8).

**Fig. 9:**
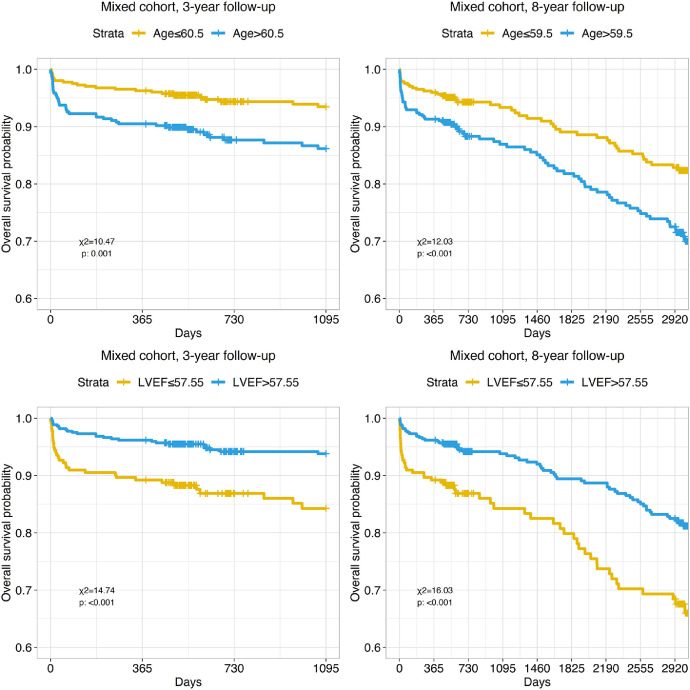
Kaplan-Meyer curves present impact of Age and left ventricle ejection fraction (LVEF) on long-term mortality in mixed patient cohort during 3 and 8-year follow-up. Cut-off values were obtained by ROC-analysis.

Kaplan-Meyer survival curves were also drowned for age, left ventricle ejection fraction, intensive care unit stay, cardiopulmonary bypass time and aortic cross-clamp time, and preoperative plasma C-reactive protein and albumin levels using cut-off values obtained by the aforementioned ROC analysis. Significant differences in survival were found for groups stratified by age and left ventricle ejection fraction (Fig. 9).

**Fig. 10:**
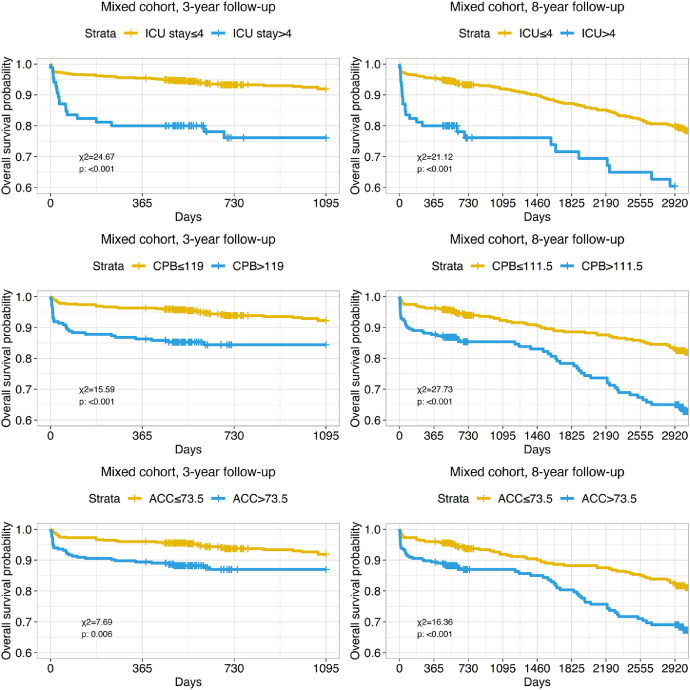
Kaplan-Meyer curves present impact of intensive care unit (ICU) stay, cardiopulmonary bypass time (CPB) and aortic cross-clamp time (ACC) on long-term mortality in mixed patient cohort during 3 and 8-year follow-up. Cut-off values were obtained by ROC-analysis.

All studied perioperative clinical characteristics (intensive care unit stay, cardiopulmonary bypass time, and aortic cross-clamp time and prolonged ICU stay more than four days) showed significant differences in survival for any observed time periods (Fig. 10).

**Fig. 11:**
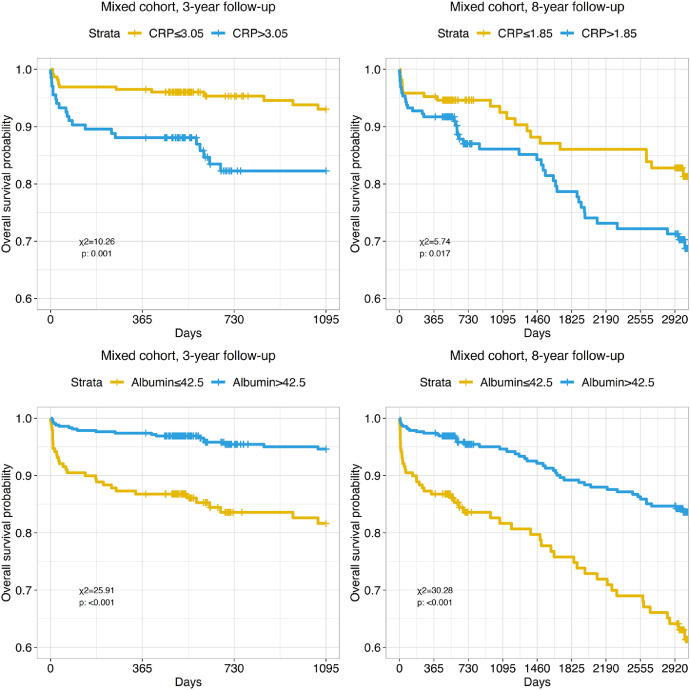
Kaplan-Meyer curves present impact of C-reactive protein and albumin on long-term mortality in mixed patient cohort during 3 and 8-year follow-up. Cut-off values were obtained by ROC-analysis.

Preoperative plasma C-reactive protein and albumin levels also showed significant differences of survival in corresponding stratified groups in 3- and 8-year follow-up (Fig. 11).

## 2 Experimental Design, Materials and Methods

### 2.1 Background

This data article presents secondary analysis of the 8-year survival among patients who were enrolled in a prospective observational study of nutritional screening in cardiac surgery. This study was approved by the local ethics committee and registered in clinicaltrials.gov (NCT01366807).

A total of 1210 eligible patients hospitalized between January 1, 2011, and August 31, 2011 were enrolled to the trial and passed nutritional screening was performed. The inclusion criteria were as follows: (1) older than 18 years and (2) elective cardiac surgery under CPB. The exclusion criteria were as follows: (1) emergency surgery, (2) aortic surgery performed under deep hypothermic circulatory arrest, (3) pulmonary thromboembolism requiring thrombectomy, and (4) off-pump surgery. The following patients were excluded: those operated without CPB (*n* = 15) and those who underwent surgery under deep hypothermic circulatory arrest (*n* = 8). Thus, the data of 1187 patients were used. Both electronic medical records and phone interviews were used for survival data collection. Long-term follow-up was conducted by phone interview.

### 2.2 Data analyses

The original dataset contains 1187 observations (rows) and 40 variables (columns). Part of lost-to-follow-up observations (449 rows) were dropped during statistical analysis.

The initial goal was determination of cut-off point for the purpose of further converting the numeric variable to factorial type. ROC analysis was performed for the following variables: Preoperative albumin level (g/l), Preoperative C-reactive protein level (mg/l), Cardiopulmonary bypass time (min), Aortic cross clamp time (min), Preoperative thrombocytes level (x10^9^/l), Left ventricle ejection fraction (%), Age (years) as predictors of death in 8 and 3 years periods of follow-up in mixed, CAD, and HVD cohorts. The target value for cut-off point was determined by selecting maximal Youden's J statistic (sensitivity + specificity – 1), corresponding value of numeric variable was used as the threshold for converting variables into two-level factor variables (low/high, with/without risk). For every ROC curve, AUC was determined using a statistical package function. P-value for AUC was determined using the Wilcoxon-Mann-Whitney U-Statistic test.

Factor variables obtained by ROC-analysis were used for stratification observation in survival analysis. For survival analysis, Kaplan-Meier models were fitted. Comparison of two cohorts was performed by log-rank test, Chi-squared statistics, and corresponding p-values were computed.

Analysis of predictors of mortality was performed via a Cox proportional hazard model. Initially, univariate models were fitted for the following variables: Logistic Euroscore, MUST>0, Cardiopulmonary bypass time (min)>Criterion, Preoperative C-reactive protein level (mg/l)>Criterion, Preoperative albumin level (g/l) > Criterion (Criterion is a corresponding value obtained by ROC analysis). Fitting multivariate models was performed by including all predictors in the model; the optimal model was obtained by step-by-step elimination of predictors with high (more than 0,05) p-values.

Statistical analysis was performed using R programming language (version 4.0.2) in the interactive development environment, RStudio (version 1.2.5001). In addition to standard packages for analysis, additional packages were also used (survival, survminer, readxl, dplyr, knitr, pROC, ggplot2, ggthemes).

## Ethics Statement

All study participants provided informed consent, and the study design was approved by the appropriate ethics review board.

## CRediT Statement

Sergey M. Efremov: Conceptualization, Methodology, Data curation, Writing - Review & Editing, Project administration. Tatiana I. Ionova: Data curation, Formal analysis. Tatiana P. Nikitina: Data curation, Formal analysis. Pavel E. Vedernikov: Investigation, Writing- Original draft preparation. Timur A. Dzhumatov: Investigation. Timofey S. Ovchinnikov: Investigation. Abduvahhob A. Rashidov: Investigation. Alexandr E Khomenko: Data curation, Formal analysis, Visualization, Writing- Original draft preparation. Christian Stoppe: Supervision. Daren K. Heyland: Supervision. Vladimir V. Lomivorotov: Supervision, Writing - Review & Editing.

## Declaration of Competing Interest

The authors declare that they have no known competing financial interests or personal relationships which have, or could be perceived to have, influenced the work reported in this article.
